# Spectrum subtraction as a complementary method for six resolution techniques resolving overlapping spectra; application to multicomponent veterinary formulation with greenness and whiteness assessment

**DOI:** 10.1186/s13065-023-01009-x

**Published:** 2023-08-15

**Authors:** Mahmoud G. Hagag, Ahmed M. Hemdan, Ahmed H. Nadim, Samah S. Abbas, Nesma M. Fahmy

**Affiliations:** 1 Analytical Chemistry Department, Faculty of Pharmacy, Ahram Canadian University, 6 October City, 4th Industrial Zone, Banks Court Street, Giza, Egypt; 2 Analytical Chemistry Department, Faculty of Pharmacy, Cairo University, El-Kasr El-Aini Street, Cairo, 11562 Egypt

**Keywords:** Green analytical chemistry, Spectrum subtraction, Factorized response method, Concentration value method, Levamisole HCl, Triclabendazole

## Abstract

Mathematical filtration is an efficient tool to resolve the overlapping spectra of binary mixtures in zero or first order form. Herein, a comparative study was conducted between six economic, accurate and precise spectrophotometric methods for determination of Triclabendazole (TCB) and Levamisole HCl (LVM). Each component was resolved with minimum mathematical steps in its zero-order absorption spectrum by ratio subtraction, constant multiplication, and the recent factorized response method; coupled with spectrum subtraction. In addition, the mixture was resolved in its first derivative form by derivative subtraction, D^1^ constant multiplication, and the recent D^1^ factorized response method; coupled with spectrum subtraction. Results obtained were also compared to those obtained from constant value, concentration value, and derivative ratio methods. The linearity range was found to be either 1.0–10.0 µg/mL or 2.0–20.0 µg/mL for TCB, and 2.0–14.0 µg/mL for LVM with LOD of 0.08 µg/mL and 0.19 µg/mL, respectively. Validation of the proposed methods was performed according to VICH guidelines. Results obtained from the statistical data showed no significant difference regarding accuracy and precision compared to the reported methods. The developed spectrophotometric methods followed the principles of green analytical chemistry, in which the green assessment was done through four tools, called, National Environmental Methods Index (NEMI), Analytical Eco-Scale (AES), Green Analytical Procedure Index (GAPI) and Analytical greenness metric (AGREE). Also, a white assessment was performed using RGB model. The proposed methods could offer an economic alternative for the routine analysis of bulk materials and combined veterinary dosage form.

**Graphical Abstract:**

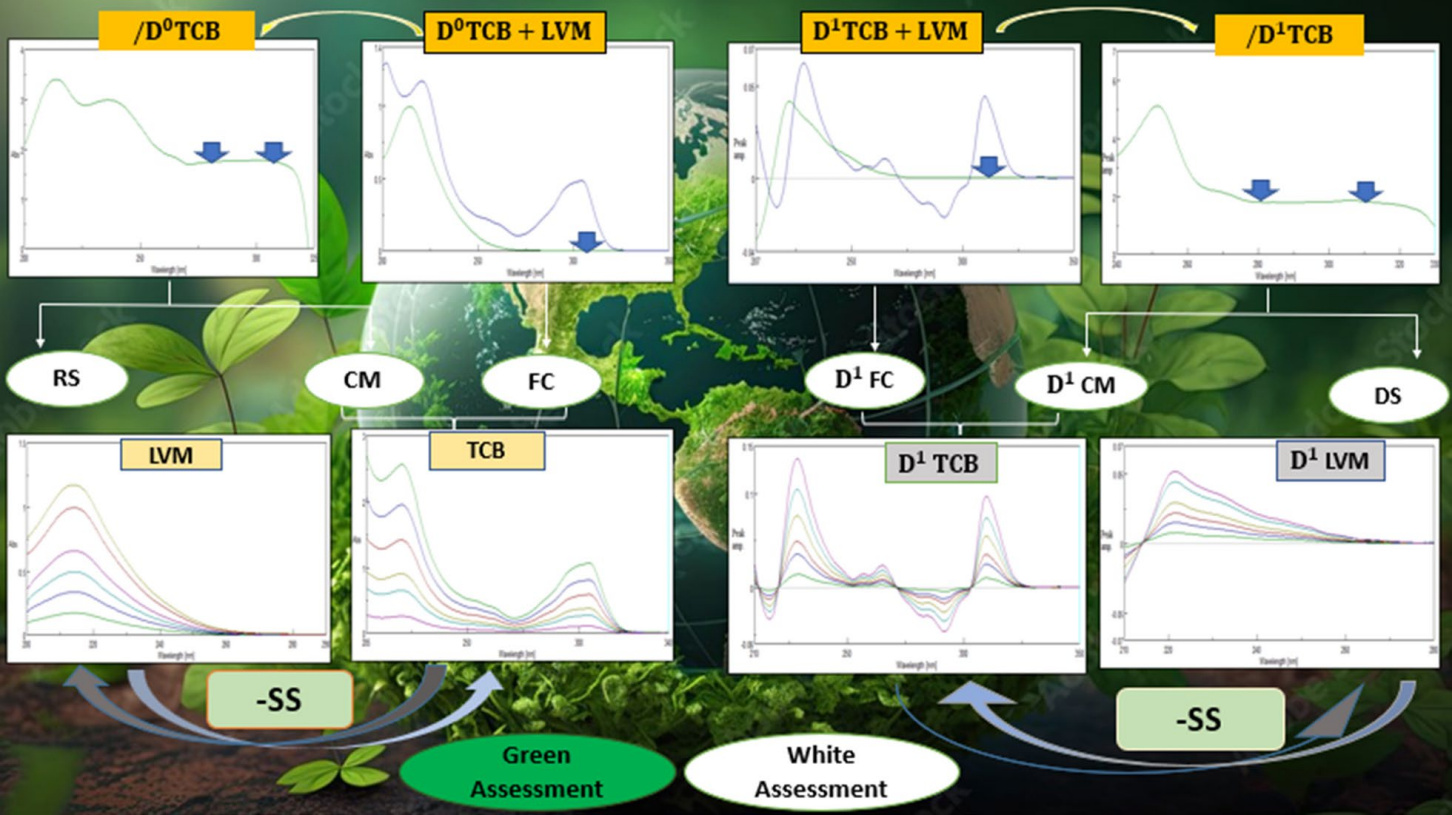

## Introduction

**Fig. 1:**
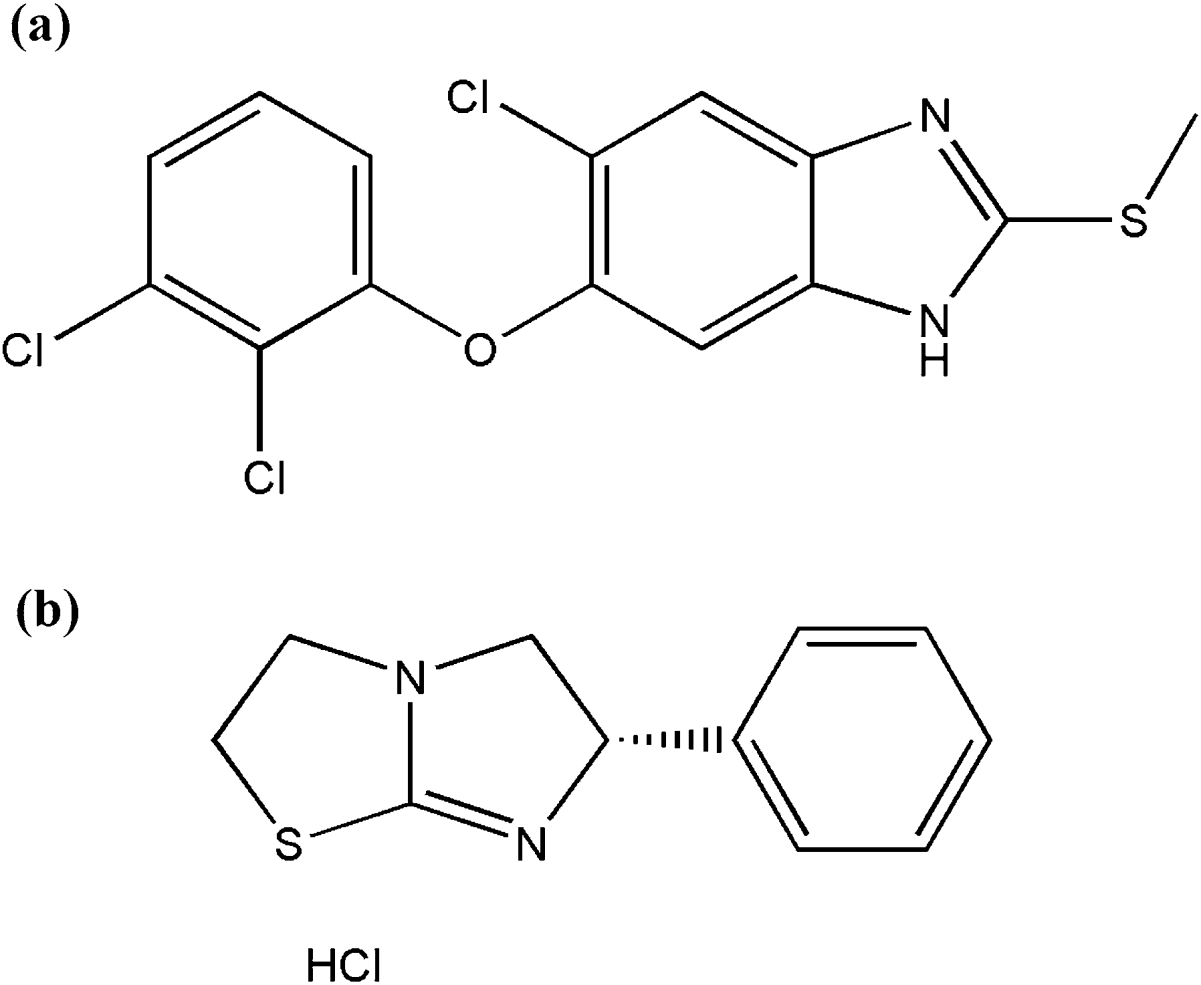
Chemical structures of (**a**) Triclabendazole, **b** Levamisole HCl

Triclabendazole (TCB),5-Chloro-6-(2,3-dichlorophenoxy)-2-(methylsulfanyl)-1H-benzimidazole is utilized regularly beginning around 1983 to treat fascioliasis in the veterinary medicine causing inhibition of microtubule formation [1, 2]. It is considered as a powerful inhibitor of the synthesis of proteins used in goat, sheep, and cattle for the treatment of fascioliasis [3]. Although US Food and Drug Administration (FDA) has not supported the use of TCB in humans, however it’s enlisted throughout many nations where fascioliasis is endemic [1, 4]. Its official Ph. Eur. method is potentiometric titration [2]. According to literature, it was reviewed in biological fluids, its metabolites presence, pharmaceutical dosage forms and pure form by spectrophotometric methods [5, 6] and chemometric methods [7, 8]. Literature also reveals its determination via HPLC [9–15] and LC–MS/ MS [16–18]. Levamisole HCl (LVM), 2,3,5,6-Tetrahydro-6-phenylimidazo[2,1-b]thiazole monohydrochloride is utilized to treat parasitic worm infection. The drug host defenses by modulating cell-mediated immune responses. It restores depressed T-cell functions than stimulating response to above-normal levels. LVM is an anthelmintic with a broad spectrum of activity which is efficient against nematodes in both mature and immature stages, as well as anthelmintic-resistant strains [19]. Its official method in Ph. Eur., USP and BP is a potentiometric titration method [2, 20, 21]. According to literature, it was reviewed in biological fluids, pharmaceutical dosage forms and pure form by spectrophotometric methods [22–24] and chemometric methods [8]. Literature also reveals its determination via HPLC [25–31], LC–MS/ MS [32, 33] and GC [34]. Both drugs chemical structures are represented in Fig. 1.

TCB and LVM mixture has been utilized especially for the treatment of acute and subacute fasciolosis outbreaks as well as the essential handling of fascicle infections. There are only two reported spectrophotometric methods for the binary mixture analysis of TCB and LVM either using chemometric tools where sophisticated software was required or derivative method in which LOD and linearity could be significantly enhanced [7, 8]. Different spectrophotometric resolution methods can be applied. Ratio subtraction method coupled spectrum subtraction (RS-SS) and derivative subtraction method coupled spectrum subtraction (DS-SS) was used to get the less extended component X through the division of the mixture spectrum with a divisor of the more extended component Y’ [35]. To get the more extended component (Y), spectrum subtraction (SS) is applied by subtraction of the X spectrum from the total mixture spectrum [36]. The same steps were repeated in DS-SS [36, 37] but on the first derivative spectrum mixture and utilizing a divisor spectrum in its first derivative, resulting in the first derivative spectra of both components allowing measurements to be done at its P_max_. Constant multiplication (CM-SS and D^1^ CM-SS) method depended on determining the concentration of Y through the division of the mixture spectrum with a divisor of Y’ (the extended component). At the extended region, the constant is measured and then multiplied by the mixture spectrum for getting the Y component (the more extended one) [37–39]. Also, spectrum subtraction method (SS) can be applied, where X could be determined by the subtraction of the Y spectrum from the total mixture spectrum [36]. The same steps were repeated in D^1^ CM-SS [36, 37] but on the first derivative spectrum mixture and utilizing a divisor spectrum in its first derivative, resulting in the first derivative spectra of both components allowing measurements to be done at its Pmax. For factorized response spectrum (FC-SS), one response value (absorbance or derivative spectrum amplitude) which corresponds to component Y where X had no contribution (λ _zero point_) using either its zero or derivative form has to be defined. The factorized response spectrum was measured by the division of (zero or derivative) every pure Y concentration by its recorded one at the selected wavelength. The spectrum of Y component obtained when the factorized spectrum is multiplied with this recorded response. By using spectrum subtraction (SS) method, X spectrum obtained by subtracting the Y spectrum from the total mixture spectrum [40–43]. For derivative ratio (DD^1^), in which there were severely overlapping spectra of the D^0^ absorption of the cited components. Then by using a divisor of X, the amplitude of derivative ratio spectra mixture was directly proportional to the concentration of Y showing no X interference. Likewise, X can be determined by using a divisor of Y [44]. Constant value (CV) is one of the constant center method’s two complementary steps [36, 38, 45] and it was utilized recently to analyze binary mixtures [39, 46]. It depended on the Y analysis in their binary mixture where X is less extended than Y. Through the division with a divisor spectrum, a constant from the plateau region parallel to the x-axis was obtained. The substitution process was done by utilizing its regression equation showing correlation between the amplitudes of ratio spectra Y/ Y’ at extended region. The corresponding concentration of Y at zero contribution of X, the Y concentration was determined. Concentration value (CNV) is a recent approach dealing with graphical manipulation of the spectra as the drug concentration value was determined directly indicating the actual concentration without any need of the substitution in its regression equation. It was performed through the division of D° mixture spectrum with the D° normalized spectrum (the more extended component). Then, the more extended component concentration was obtained from the constant determined from the plateau region parallel to the x-axis. [47, 48].

The aim of the current study was to establish a comparable study to develop six simple, economical, sensitive spectrophotometric methods which have the ability to resolve the spectral overlap of TCB and LVM in its dosage form. This work has been enhanced regarding the safety of humans and the environment due to the green ecofriendly methods which are safe for the planet also the sustainability ensured by white chemistry so it will not harm the future generations. This should offer a cost-effective alternative for the analysis of veterinary formulations.

## Experimental

### Device and software

A double-beam UV/Visible spectrophotometer model V-760 (Jasco, Japan) was used in spectrophotometric measurements. Data acquisition was done using Spectra manager^®^ software JASCO corporation version 2 (Japan). The quartz cells used were obtained from Chromtech (UK) with 45 mm × 12.5 mm × 12.5 mm, 3.5 mL (1.0 cm)—2 optical faces. The absorption spectra of the blank and sample solutions were tested over 200—400 nm range.

### Chemicals and reagents

#### Materials and reagents

Pure samples of TCB and LVM were provided from Pharma Swede company, Egypt with purity of 99.88 ± 0.84 and 100.32 ± 1.44 respectively, in accordance with Ph. Eur. official methods [2]. Eradex Forte Suspension^®^ each 1 mL of consists of 120 mg TCB and 75 mg of LVM was also obtained from Pharma Swede company, Egypt. Methanol and HCl of analytical grade were purchased from Piochem company, Egypt.

#### Standard stock solutions

Standard stock solutions were made to contain 500.0 µg/mL of each, TCB and LVM in methanolic HCl solution. Working standard solutions were prepared to contain 50.0 µg/mL of each, TCB and LVM in methanolic HCl solution. Throughout the lab work, standard stock solutions were kept in refrigerator (2–8 °C) for up to 5 days in glass-stoppered volumetric flasks.

### Procedure

#### Spectral characteristics

Through scanning 200–400 nm range against methanolic HCl as a blank, the D^0^ absorption spectra of 10.0 µg/mL TCB and 10.0 µg/mL LVM were obtained. Then, by spectra manager^®^ software, the derivatized spectra were obtained and kept on the computer.

#### Factorized spectra of TCB

The D^0^ TCB spectrum was divided by the recorded absorbance at 304 nm and saved on the computer. The D^1^ TCB spectrum was divided by the recorded amplitude at 310 nm and saved on the computer.

#### Linearity and calibration graphs:

Portions equivalent to 10.0–200.0 µg TCB and 20.0–140.0 LVM were transferred from the working solutions (50.0 µg/mL) for TCB and LVM, into two volumetric flasks series of (10-mL) and completed to volume with methanolic HCl for TCB and LVM. The prepared standards spectra were scanned in the range of 200–400 nm against a blank then stored on the computer.

##### Calibration graphs for the zero order absorption spectra (D^0^) of TCB and LVM

Regression equations showing the linearity of relationships between the absorbance at λ_max_ of the scanned spectra of TCB at 304 nm and 220 nm, and of LVM at 214 nm, versus the corresponding TCB or LVM concentrations were calculated.

##### Calibration graphs for the first derivative (D^1^) spectra of TCB and LVM

The first derivative spectra were determined using ∆λ = 4, and a scaling factor = 10. For obtaining the regression equations, calibration graphs were performed relating the peak amplitude (P-Zero) of the D^1^ spectra of TCB at 310 nm and at P _max–min_ (P _228–216_) and of LVM at P _max_ (P _221 nm_) versus the corresponding concentrations.

##### Calibration graphs for the first derivative of ratio spectra (DD^1^) of TCB and LVM

Regression equation relating amplitudes of the ratio spectra D^1^ of TCB at P _max–min_ (P_230-220 nm_) and LVM P _max_ (P 219 nm) versus the concentrations were calculated.

##### Calibration graphs for constant value method (CV)

D^0^ TCB spectra was divided by a divisor of normalized TCB. For computing the regression equation, the calibration graph was performed by plotting amplitude at the plateau region 290–306 nm versus the corresponding concentration of TCB.

#### Applying the spectrophotometric methods for determining TCB and LVM in laboratory prepared mixtures

**Scheme 1:**
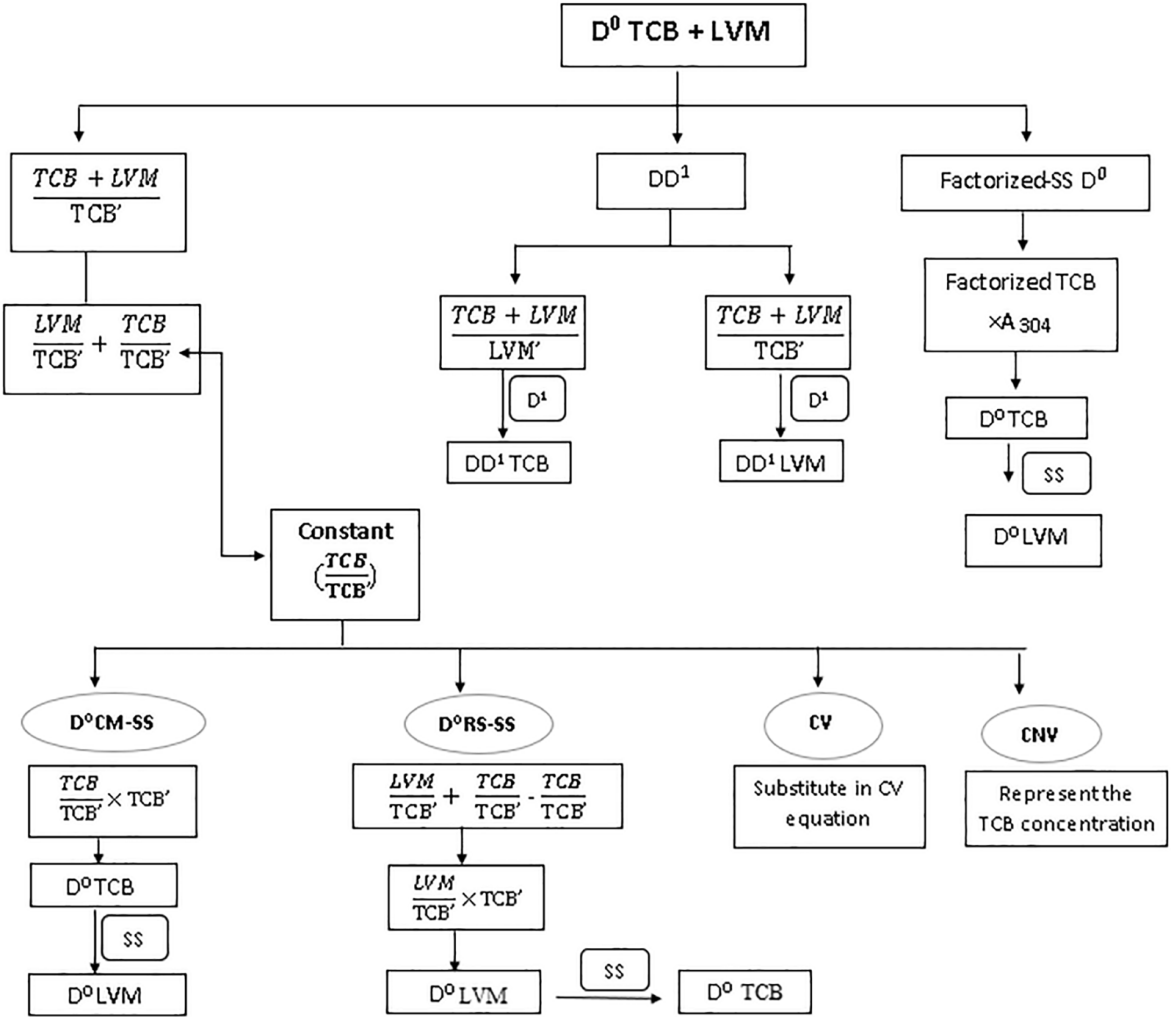
Methods applied on D^0^ for Triclabendazole and Levamisole HCl mixture

**Scheme 2:**
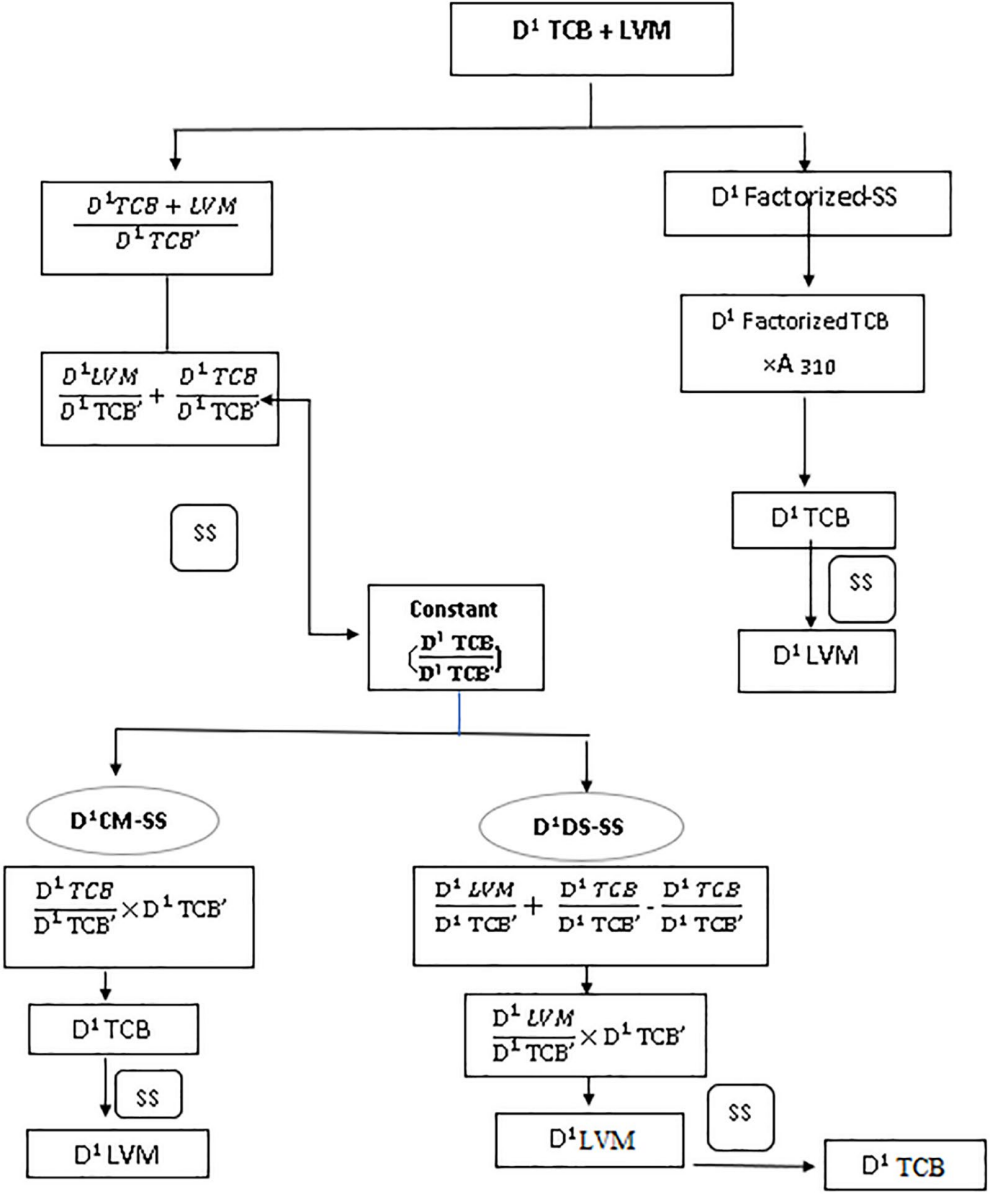
Methods applied on D^1^ for Triclabendazole and Levamisole HCl mixture

TCB and LVM aliquots were transferred from their working solutions separately. Methanolic HCl was added to achieve the needed volume and obtain mixtures of various ratios of drugs under study and proceeded to determine the binary mixture concentration of TCB and LVM. Through the substitution of the absorbance of TCB at 304 nm in its corresponding regression equation, TCB concentration was obtained. Then, steps in scheme 1 were applied. Also, from the first derivative form of the lab mixture; for having TCB concentration, the peak amplitude at 310 nm was substituted in the corresponding regression equation. Then, the steps in scheme 2 were applied.

##### Ratio subtraction method coupled spectrum subtraction (RS-SS) and derivative subtraction method coupled spectrum subtraction (DS-SS)

Through the division of the mixture D^0^ absorption spectrum by TCB’ divisor D^0^ spectrum of 5.0 µg/mL, the constant (TCB/TCB֙) was obtained. This constant was subtracted from the D^0^ binary mixture spectrum followed by multiplication with D^0^ TCB’ spectrum divisor of 5.0 µg/mL to get the less extended D^0^ LVM spectrum to measure the absorbance as its λ_max_ 214 nm. The D^0^ TCB more extended spectrum was produced by subtraction of the resolved D^0^ spectrum of LVM from the D^0^ binary mixture spectrum, allowing measurement of the absorbance at its λ_max_ 304 nm and 220 nm.

Through the division of the mixture D^1^ derivative spectrum by TCB’ divisor D^1^ spectrum of 5.0 µg/mL, the constant (TCB/TCB֙) was obtained. This constant was subtracted from the D^1^ binary mixture spectrum followed by the multiplication with D^1^ TCB’ spectrum divisor of 5.0 µg/mL to get the less extended D^1^ LVM spectrum allowing measurement of the absorbance at its P_max (P 221 nm)._ The D^1^ TCB more extended spectrum was produced by subtraction of the resolved D^1^ spectrum of LVM from the D^1^ binary mixture spectrum, allowing measurement of the amplitude at its P (310 nm) and P _max–min_ (P 228–216).

##### Constant multiplication (CM-SS and D.^1^ CM-SS)

Through the division of the mixture D^0^ absorption spectrum by TCB’ divisor D^0^ spectrum of 5.0 µg/mL, the constant (TCB/TCB֙) was obtained. The constant value in the extended region (290–306 nm) was reported and then multiplied with the 5.0 µg/mL TCB spectrum divisor to attain resolved D^0^ more extended spectrum of TCB. Then, the absorbance at 220 nm in its corresponding regression equation was substituted. The D^0^ less extended spectrum of LVM was obtained when the resolved D^0^ TCB spectrum was subtracted from D^0^ total mixture spectrum. Then, the absorbance at 214 nm in its corresponding regression equation was substituted.

Through the division of the mixture D^1^ absorption spectrum by TCB’ divisor D^1^ spectrum of 5.0 µg/mL, the constant (TCB/TCB֙) was obtained. The constant value in the extended region (280–315 nm) was reported and then multiplied with the D^1^ TCB spectrum divisor of 5.0 µg/mL to attain resolved D^1^ more extended TCB spectrum. Then, the absorbance at P _max–min_ (P 228–216) in its corresponding regression equation was substituted. From total mixture spectrum, when the resolved D^1^ TCB spectrum was subtracted from the D^1^ binary mixture spectrum, it led to the formation of the D^1^ less extended spectrum of LVM. Then, the absorbance at P _max_ (P 221) nm was substituted in its corresponding regression equation.

##### Factorized response spectrum (D^0^ FC-SS and D.^1^ FC-SS)

The D^0^ TCB was determined through the multiplication of the peak amplitude at 304 nm with the TCB factorized spectrum λ_max_ at 220 nm. Then subtracted from the mixture, to get LVM which was measured at 214 nm. The peak amplitude at 310 nm was multiplied with the TCB factorized spectrum after derivatization to get its D^1^ spectrum at P _max–min_ (P _228–216 nm_). Then subtracted from the D^1^ binary mixture spectrum to get the D^1^ of LVM which have P _max_ (P _221 nm_).

##### Derivative ratio (DD^1^) of TCB and LVM

The ratio spectra first derivative of TCB using LVM divisor of 4.0 µg/mL was determined, where the amplitude at P _max–min_ (P _230–220 nm_) was substituted in the corresponding regression equation to obtain TCB concentration. The ratio spectra first derivative of LVM using TCB divisor of 3.0 µg/mL was determined and the amplitude at P _max_ (P _219 nm_) was substituted in the corresponding regression equation to obtain LVM concentration.

##### Constant value (CV)

The same constant derived from the plateau region 290–306 nm was substituted directly in CV regression equation at 304 nm to obtain TCB concentration.

##### Concentration value (CNV)

From D^0^ spectrum of TCB, the peak amplitude was determined on the spectrum chart at plateau region 290–306 nm then R% was calculated.

#### Application to pharmaceutical formulation

One mL (1.045 is an average wt/mL) of Eradex Forte suspension^®^ was transferred to volumetric flask (100-mL) and 50-mL of a solvent mixture of methanol: HCl (90:10, v/v) was added. The solution was then shaken for 5 min. The solution was sonicated for at least 40 min. and then filtered 3 times. The filtrate was completed with the same solvent mixture of methanolic HCl to the mark to get a stock solution with claimed concentrations of 1200.0 and 750.0 µg/mL TCB and LVM, respectively. One mL of the stock solution was transferred into 50-mL volumetric flask and the volume was completed to the mark with methanolic HCl to have a final concentration of working solution 24.0 μg/mL and 15.0 μg/mL for TCB and LVM, respectively. As mentioned under analysis of laboratory prepared mixtures, the assay was done.

## Results and discussion

In the previous years, several resolution techniques were proposed to mathematically filter the spectrum of each component in binary mixture in either its D^0^ or D^1^ absorption spectrum. The resolution techniques such as ratio subtraction or derivative subtraction could resolve the less extended component spectrum, while methods such CM and extended ratio subtraction (EXRS) could resolve the spectrum of the more extended component [46]. Combining two techniques such as RS/EXRS [47], or RS/CM [40] could resolve separately each component in the binary mixture. But as each method was composed of many mathematical steps, spectrum subtraction proved to be the best complementary method to any resolution technique able to resolve one component separately from the binary mixture, with the least manipulation steps and hence highest accuracy, eliminating any error that might appear from the choice of divisors and measurements at critical points.

**Fig. 2:**
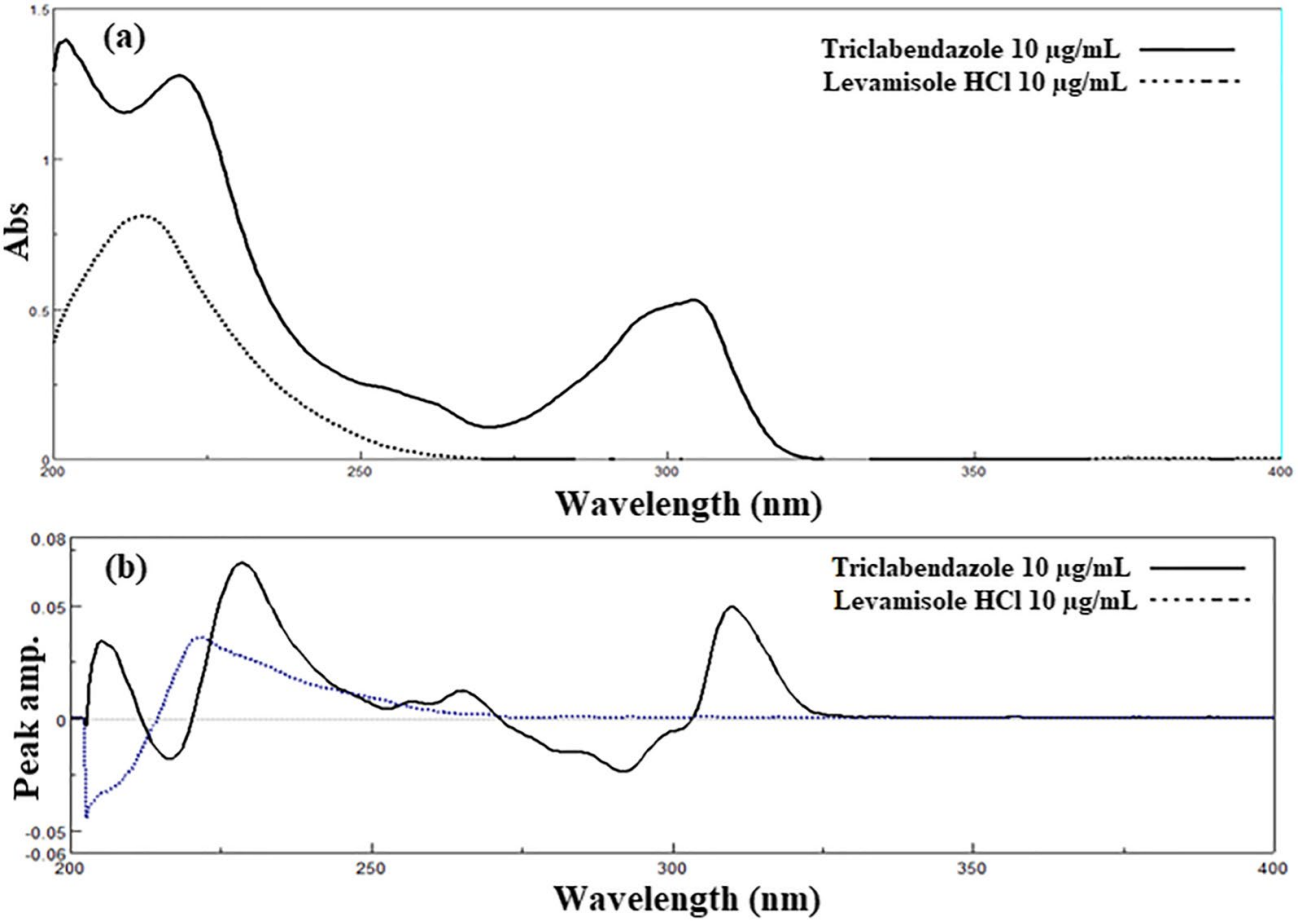
**a** The zero order absorption spectra of Triclabendazole and Levamisole HC **b** The first derivative absorption spectra of Triclabendazole and Levamisole HCl

As shown in Fig. 2a, TCB could be determined at its λmax 304 nm where LVM had no contribution. But by applying resolution techniques that resulted in TCB zero order absorption, spectrum filtered alone allowed measurements to be done at its higher sensitivity peak 220 nm and eliminating the interference from LVM. Similarly, Fig. 2b shows that TCB could be determined directly at its P max 310 nm. With applying resolution techniques that resulted in the first derivative form of TCB alone allowed measurements to be conducted at Pmax-min (P _228–216 nm_) with higher sensitivity and eliminating the interference from LVM.

In this study, SS was coupled with six techniques, namely RS, DS, D^0^ CM, D^1^ CM, D^0^ FC, and D^1^ FC resolution techniques. A comparative study was established among all the proposed resolution techniques and the results were compared to DD^1^, CV, and CNV methods. The absorption spectrum of the two drugs under investigation showed high overlapping as indicated in Fig. 2. The TCB spectrum was more extended than LVM in the wavelength region from 290–306 nm. TCB could be determined directly at a λ_max_ 304 nm. As presented in Fig. 2, TCB had 2 peaks at 220 nm with linearity range 1.0–10.0 µg/mL and at 304 nm with linearity 2.0–20.0 µg/mL. Since the peak at 220 nm had higher sensitivity, several spectrophotometric methods were conducted to allow measurements at this λ_max_. Six resolutions techniques were conducted to obtain both TCB and LVM separately in their zero-order spectrum or first derivative absorption spectra, allowing measurement of each drug at its robust λ_max_ or P_max_ with highest accuracy and precision.

### Ratio subtraction (RS-SS) and constant multiplication (CM-SS)

The first resolution technique succeeded in producing the D^0^ of LVM which is the less extended component was ratio subtraction (RS). This allowed measurements to be conducted at its λ_max_ 214 nm. By the application of spectrum subtraction (SS), the more extended D^ο^ TCB spectrum was obtained when the less extended D^0^ of LVM was subtracted from the D^0^ of the mixture spectrum, letting measurements of TCB to be applied at its λmax 304 nm and 220 nm. Thus, RS/SS succeeded to obtain the spectra of each component alone in its D^0^ absorption spectrum.

**Fig. 3:**
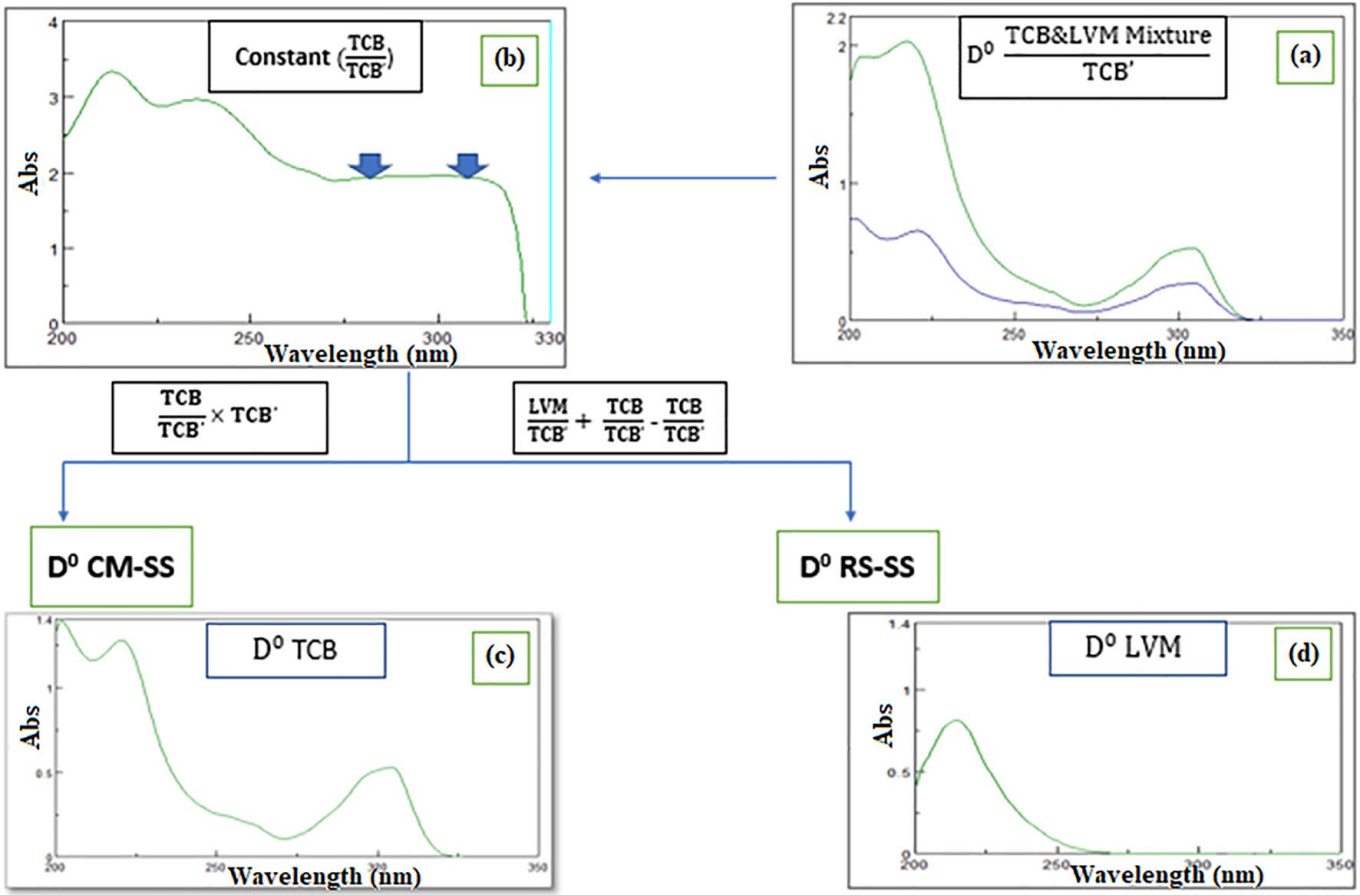
**a** The zero order absorption spectrum of Triclabendazole and Levamisole HCl mixture, 10 µg/mL of each overlaid with 5.0 µg/mL triclabendazole as a divisor **b** The ratio spectrum of the mixture of Triclabendazole and Levamisole HCl divided by 5.0 µg/mL triclabendazole as a divisor **c** The resolved D^0^ absorption spectrum of Triclabendazole **d** The resolved D^0^ absorption spectrum of Levamisole HCl

Constant multiplication was the resolution technique that succeeded in obtaining the more extended component D^0^ spectrum of TCB, letting detections to be conducted at its λmax 220 nm. Then by coupling this method with SS, the less extended D^0^ LVM spectrum was obtained, where the resolved D^0^ TCB spectrum was subtracted from the D^0^ total mixture spectrum, allowing its measurement at its λ_max_ 214 nm. Thus, CM/SS succeeded to obtain the spectra of each component alone in its zero-order absorption spectrum D^0^ as indicated in Fig. 3.

### Factorized spectrum (FC-SS)

**Fig. 4:**
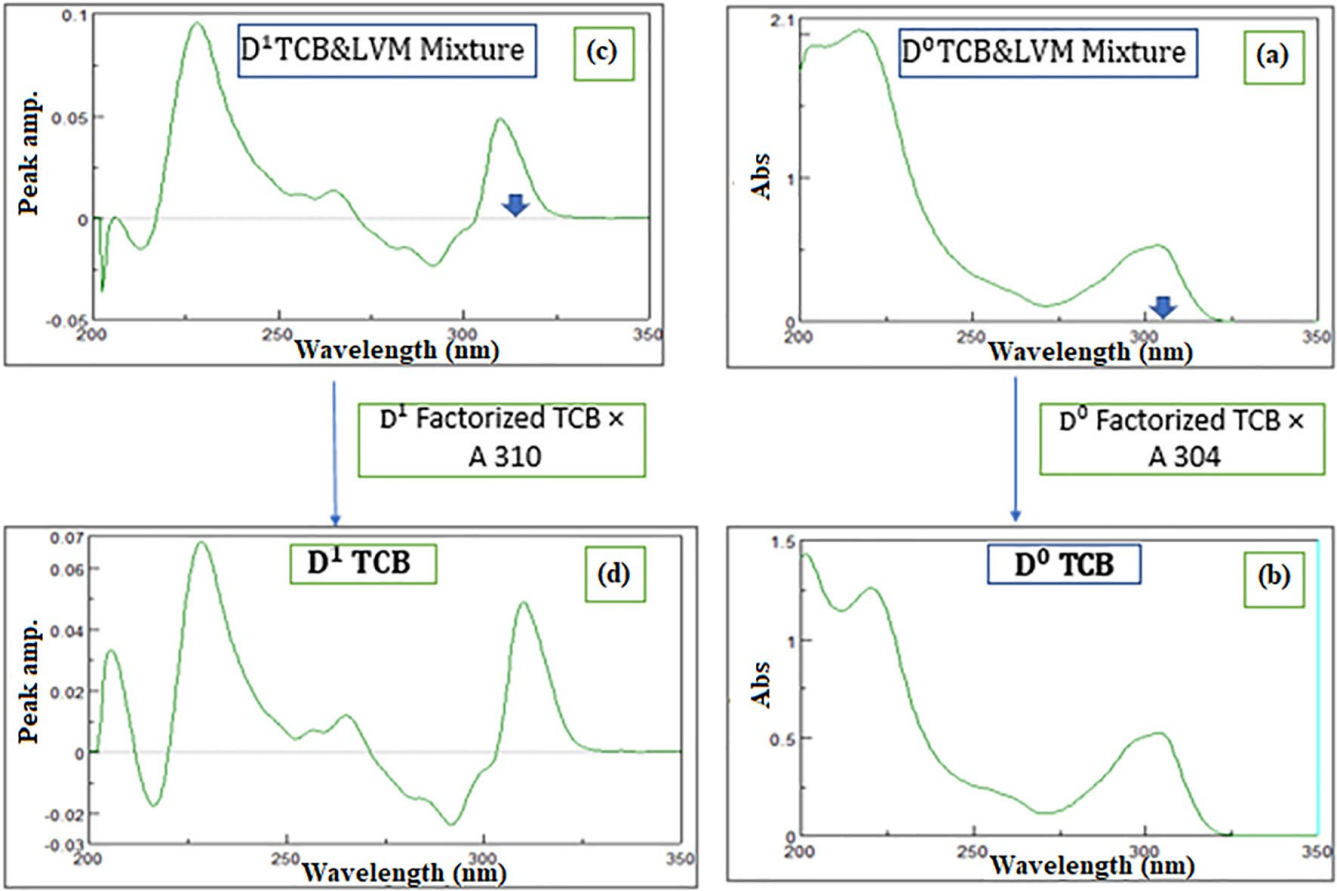
**a** The zero-order absorption spectrum of Triclabendazole and Levamisole HCl mixture, 10 µg/mL **b** The resolved D^0^ absorption spectrum of Triclabendazole **c** The first order absorption spectrum of Triclabendazole and Levamisole HCl mixture,10 µg/mL **d** The resolved D^1^ absorption spectrum of Triclabendazole

D^ο^ Factorized resolution technique succeeded in having the D^0^ of the more extended component TCB at its λ_max_ 220 nm. Coupling this method with SS succeeded in obtaining the D^0^ of the less extended component LVM letting measurements to be conducted at its λ_max_ 214 nm. Thus, D^ο^ FC/SS succeeded to obtain the spectra of each component alone in its D^0^ absorption spectrum as shown in Fig. 4.

Both RS and CM depended upon using a divisor of the extended component, while D^ο^ factorized resolution technique depended upon using a single wavelength where LVM had no contribution, and without the use of any divisors. Although RS/SS. D^0^ CM/SS, and D^0^ FC/SS ended up in resolving each component in its zero-order absorption spectrum, but D^ο^ FC/SS proved to be the most time effective technique, with minimum manipulation steps and no divisor. Comparing RS/SS with D^0^ CM/SS where both deal with using a divisor of the extended component, where a constant was acquired from the plateau region parallel to the x-axis. In CM, the constant was multiplied by the divisor for obtaining D^0^ spectrum of TCB and coupling it to SS the LVM spectrum was obtained. In RS, the constant was first subtracted from the mixture’s ratio spectrum then, multiplied with the divisor spectrum to get the less extended component LVM and coupling it to SS the TCB spectrum was obtained. The fewer steps in CM/SS made it superior to RS/SS.

### Derivative subtraction (DS-SS), constant multiplication (D^1^ CM-SS) and factorized spectrum (D.^1^ FC-SS)

The three techniques applied on the zero order absorption spectra were repeated on the first derivative spectra mixture showing each component first derivative spectrum alone where LVM was measured at its P_max_ 221 nm and TCB was measured at its P_max-min_ (228–216 nm). The first derivative spectra of both components showed a wider extended region (280–315 nm) where LVM had no contribution. Derivative subtraction (DS) resulted in the first derivative of the less extended component LVM and when coupled to SS, the extended component D^1^ spectrum of TCB was obtained. D^1^ CM succeeded in obtaining the D^1^ spectrum of the extended component TCB and when coupled to SS, the D^1^ spectrum of the less extended component LVM was obtained. Both DS/SS, and D^1^ CM/SS generated the first derivative spectrum of each component depending on a D^1^ 5.0 µg/mL TCB divisor. But, D^1^ CM/SS showed fewer manipulation steps. The third resolution technique D^1^ FC/SS was superior to the previous two techniques and succeeded to obtain the first derivative spectrum of each component with the least manipulation steps.

### Constant value (CV) and concentration value (CNV) methods

**Fig. 5:**
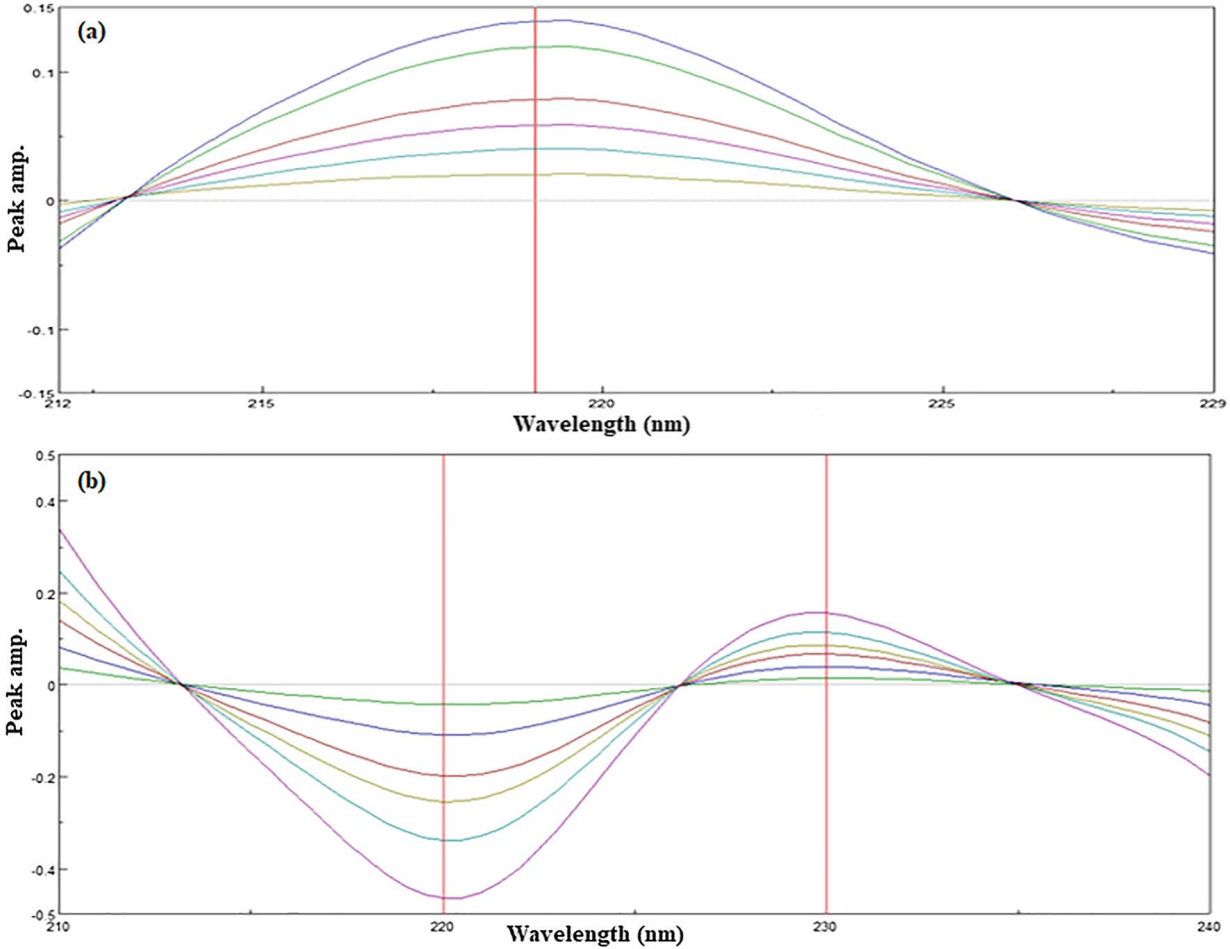
**a** The first derivative spectra of ratio spectra of Levamisole HCl (2.0–14.0 µg/mL) using 3.0 µg/mL triclabendazole as a divisor **b** The first derivative of ratio spectra of Triclabendazole (2.0–20.0 µg/mL) using 4.0 µg/mL Levamisole HCl as a divisor

The same constant that resulted from the division of the lab mixture spectrum by a divisor of normalized TCB’ was either substituted in the corresponding regression equation to have TCB concentration by CV method or was directly represented the TCB concentration depending upon the CNV method. Due to the severely overlapping spectrum of both components in the wavelength region (200–250 nm), DD^1^ resolution technique methods in which [44] using LVM divisor of 4.0 μg/mL, the LVM contribution in the lab mixture is cancelled when the ratio spectrum is derivatized, and the peak amplitude at P _max–min_ (P _230–220 nm_) is directly proportional to the TCB concentration. While using 3.0 μg/mL TCB as a divisor, the TCB contribution in the lab mixture is cancelled when the ratio spectrum is derivatized and the peak amplitude at P _max_ (P 219 nm) is directly proportional to the LVM concentration as shown in Fig. 5.

### Application to pharmaceutical formulation

This study was performed on Eradex Forte suspension^®^ dosage form which contains two components of TCB and LVM. According to literature, TCB was soluble in many methanolic solvents. Methanolic HCl was proven to be the best solvent which gave the most sensitive peak [5]. The literature had ensured that LVM was freely soluble in 0.1N HCl [22], so methanolic HCl was the solvent of choice. Many trials were performed to choose the best solvent ratio of methanol: HCl (50:50, 60:40, 70:30, 80:20, and 90:10). The ratio of methanol: HCl; 90:10 was chosen as the best ratio to extract the dosage form under study. Ultra-sonication time required for dosage form extraction was tried after 20, 25, 30, 35, and 40 min to get the best clear solution. The 40 min ultra-sonication proved to give satisfactory results. It should be noted that the proposed methods proved their sensitivity and selectivity in obtaining the spectra profile of TCB and LVM compared to literature [7, 8]. They spared the need of any sophisticated manipulation software. Also, the study had showed improved LOD for TCB and LVM of 0.08 and 0.19 µg/mL, respectively compared to 0.14 and 0.52 µg/mL for the reported method. Additionally, these methods successfully proved their safety to human and environment. The methods are cost effective as well as can be utilized easily in the regular analysis of QC labs for drugs used in cattle breeding industry.

## Validation

In compliance with VICH guidelines, methods validation was developed [49], where the parameters were presented in Table 1. They showed good accuracy, precision, and sensitivity. The specificity was evaluated through the binary mixtures analysis including a variety of drug ratios, and the methods were shown to be specific as indicated in Table 2. The developed methods were successfully used in the assay of cited drugs in Eradex Forte suspension^®^. Also, the results were statistically compared to reported methods as indicated in Table 3 and 4. Table 5 displayed the statistical results obtained by comparing the proposed methods to the reported method which show no significant difference.

**Table 1:** Assay parameters and method validation obtained by applying the proposed spectrophotometric methods

Drug Name	TCB							LVM		
Method	D^0^ (304 nm)	D^0^ (220 nm)	D^1^ (310 nm)	D^1^ (P_228–216 nm_)	DD^1^ (P_230–220 nm_)	CNV	CV	D^0^ (214 nm)	D^1^ (P_221 nm_)	DD^1^ (P_219 nm_)
Range (µg/mL)	2.0–20.0	1.0 – 10.0	2.0–20.0	1.0–10.0	2.0–20.0	…	2.0–20.0	2.0–14.0		
Slope	0.0533	0.1322	0.0049	0.0088	0.0306	…	1.0043	0.0834	0.0037	0.0099
Intercept	0.0011	− 0.0198	0.0001	− 0.0002	− 0.0031	…	0.0177	− 0.003	− 0.0002	− 0.0004
Accuracy (R% ± SD)	100.78 ± 0.61	101.05 ± 0.88	100.84 ± 0.46	100.002 ± 0.80	98.76 ± 0.59	101.01 ± 0.44	100.41 ± 0.37	99.35 ± 0.76	98.79 ± 0.94	100.54 ± 0.37
Correlation coefficient (r)	0.9998	1.0000	0.9999	0.9997	0.9999	…	0.9998	0.9997	0.9998	0.9998
Repeatability (RSD)^a^	0.176	0.522	0.334	0.410	0.585	0.100	0.184	0.537	0.365	0.203
Intermediate precision (RSD)^b^	0.554	1.159	0.386	0.531	0.821	0.216	0.259	0.565	0.383	0.769
LOD	0.28	0.08	0.26	0.14	0.52	…	0.29	0.27	0.19	0.23
LOQ	0.86	0.23	0.78	0.41	1.58	…	0.89	0.80	0.58	0.70

RSD^a^ &RSD^b^: relative standard deviation for the intra-day & inter-day precision, respectively (n = 3) using concentrations 4, 6, 9 µg/ mL for TCB and 6, 9, 13 µg/ mL for LVM

**Table 2:** Determination of TCB and LVM in laboratory prepared mixtures and in Eradex Forte^®^ suspension by the proposed methods

TCB:LVM ratio		TCB								
		D^0^(304 nm)	D^0^(220 nm)			D^1^(310 nm)	D^1^(P _228–216 nm_)			DD^1^(P_230-220 nm_)
		RS-SS	RS-SS	CM-SS	FC-SS	DS-SS	DS-SS	D^1^ CM-SS	D^1^ FC-SS	
		R% ± S.D								
**2**	**3**	98.49 ± 0.56	98.06 ± 0.05	98.01 ± 0.12	98.06 ± 0.06	101.33 ± 0.42	100.75 ± 0.41	100.80 ± 0.43	100.67 ± 0.53	99.93 ± 1.23
**3**	**4**	98.84 ± 0.33	98.18 ± 0.13	98.25 ± 0.14	98.26 ± 0.19	101.2 ± 0.34	100.28 ± 0.62	100.47 ± 0.43	99.57 ± 0.53	99.85 ± 1.29
**1**	**1**	99.86 ± 1.94	98.29 ± 1.99	100.58 ± 1.78	101.09 ± 1.94	101.18 ± 0.98	100.43 ± 0.69	100.99 ± 0.59	100.81 ± 0.49	99.99 ± 1.07
**4.5**	**2.5**	99.82 ± 0.17	98.61 ± 0.12	98.71 ± 0.21	98.54 ± 0.09	101.89 ± 0.35	100.41 ± 0.94	101.08 ± 0.96	101.53 ± 0.53	101.72 ± 0.72
*4	*2.5	98.57 ± 0.75	101.06 ± 1.97	100.3 ± 1.76	101.12 ± 1.94	100.31 ± 1.53	100.49 ± 0.62	101.81 ± 0.66	99.34 ± 0.53	99.53 ± 0.99
Eradex Forte^®^ Suspension										
R% ± S.D										
*4	*2.5	99.97 ± 1.42	100.19 ± 1.98	100.19 ± 1.99	100.26 ± 1.53	100.55 ± 1.99	100.53 ± 1.99	100.47 ± 1.98	100.58 ± 1.89	98.819 ± 0.827

TCB:LVM ratio		TCB		LVM						
		CV	CNV	D^0^(214 nm) FC-SS			D^1^(P_221 nm_)			DD^1^(P_219 nm_)
				RS-SS	CM-SS		DS-SS	D^1^ CM-SS	D^1^ FC-SS	
		R% ± S.D								
**2**	**3**	98.49 ± 0.55	99.13 ± 0.56	99.05 ± 0.89	100.12 ± 1.014	99.92 ± 1.35	101.2 ± 0.69	101.91 ± 0.41	101.76 ± 0.62	100.46 ± 0.85
**3**	**4**	98.88 ± 0.09	99.49 ± 0.09	99.31 ± 0.96	98.85 ± 1.29	98.88 ± 1.40	99.03 ± 0.89	98.96 ± 0.67	99.06 ± 0.51	99.102 ± 1.29
**1**	**1**	98.93 ± 1.83	99.61 ± 1.83	101.7 ± 0.50	101.2 ± 0.59	101.46 ± 0.97	100.77 ± 0.77	100.51 ± 0.97	100.39 ± 1.39	100.14 ± 1.44
**4.5**	**2.5**	100.11 ± 0.42	100.74 ± 0.42	101.68 ± 1.44	101.99 ± 1.35	101.76 ± 1.46	101.8 ± 0.83	100.54 ± 0.54	100.18 ± 0.83	100.61 ± 1.07
*4	*2.5	100.47 ± 1.19	101.13 ± 1.19	101.93 ± 1.07	101.48 ± 1.26	101.44 ± 0.96	100 ± 1.95	100.72 ± 1.65	100.3 ± 1.65	100.21 ± 0.94
Eradex Forte^®^ Suspension										
R% ± S.D										
*4	*2.5	99.09 ± 1.21	99.78 ± 1.32	100.25 ± 1.96	99.39 ± 1.74	100.004 ± 1.53	100.62 ± 1.94	99.24 ± 1.83	99.45 ± 1.77	101.98 ± 0.39

*Dosage form ratio

**Table 3:** Statistical comparison between results obtained by the proposed methods and the reported methods for the determination of TCB and LVM in pure powder form

	TCB								LVM			
Parameters	Reported Method^a^ [5]	D^0^ (304 nm)	D^0^ (220 nm)	D^1^ (310 nm)	D^1^ (P _228–216 nm_)	DD^1^ (P _230–220 nm_)	CV	CNV	Reported Method^b^ [22]	D^0^ (214 nm)	D^1^ (P 221 nm)	DD^1^ (P 219 nm)
Mean	99.88	99.92	99.73	100.39	99.81	99.85	99.88	100.48	100.32	100.17	99.35	100.10
S.D	0.84	0.90	0.87	0.83	0.85	1.49	1.03	1.35	1.44	1.26	0.82	1.15
n	6	6	6	6	6	6	6	6	6	6	6	6
Variance	0.71	0.81	0.76	0.69	0.72	2.22	1.06	1.82	2.07	1.59	0.67	1.32
F (5.05)	…	1.14 (5.05)	1.07 (5.05)	1.03 (5.05)	1.01 (5.05)	3.13 (5.05)	1.49 (5.05)	2.5896	…	1.30 (5.05)	3.09 (5.05)	1.57 (5.05)
Student's t^c^ (2.228)	…	0.085 (2.228)	0.29 (2.228)	1.05 (2.228)	0.14 (2.228)	0.04 (2.228)	0.007 (2.228)	0.925	…	0.19 (2.228)	1.43 (2.228)	0.28 (2.228)

^a^ Spectrophotometric method using 0.1 M methanolic HCl as a solvent at wavelength of 305 nm

^b^ Spectrophotometric method using 0.1N HCl as a solvent at wavelength of 216 nm

^c^ The values are the corresponding tabulated values of t and F at p = 0.05

**Table 4:** Statistical comparison between results obtained by the proposed methods and the reported method for the determination of TCB and LVM in Eradex Forte^®^ suspension

Parameters	TCB												LVM							
	Reported Method^a^ [8]	D^0^ (304 nm)	D^0^ (220 nm)			D^1^ (310 nm)	D^1^ (P _228–216 nm_)			DD^1^ (P_230-220nm_)	CV	CNV	Reported Method^a^ [8]	D^0^ (214 nm)			D^1^ (P_221 nm_)			DD^1^ (P_219 nm_)
		RS-SS	RS-SS	CM-SS	FC-SS	DS-SS	DS-SS	D^1^ CM-SS	D^1^ FC-SS					RS-SS	CM-SS	FC-SS	DS-SS	D^1^ CM-SS	D^1^ FC-SS	
**Mean**	99.89	99.97	100.19	100.19	100.26	100.55	100.53	100.44	100.49	98.97	99.09	99.78	100.48	100.25	99.39	100.01	100.62	99.37	99.45	102.09
**S.D**	0.59	0.59	1.98	1.99	1.53	1.99	1.99	2.05	2.04	0.62	1.21	1.32	0.76	0.76	1.74	1.53	1.95	2.07	1.77	0.58
**n**	3	3	3	3	3	3	3	3	3	3	3	3	3	3	3	3	3	3	3	3
**Variance**	0.35	0.348	3.92	3.98	3.06	3.98	3.98	4.20	4.08	0.38	1.47	2.64	0.58	0.58	3.03	2.34	3.80	4.28	3.54	1.16
**F** **(19)**	…	5.828 (19)	11.297 (19)	11.45 (19)	6.74 (19)	11.49 (19)	11.42 (19)	12.04 (19)	11.92 (19)	1.12 (19)	4.24 (19)	5.02 (19)	…	6.75 (19)	5.28 (19)	4.12 (19)	6.64 (19)	7.47 (19)	5.51 (19)	1.71 (19)
**Students t**^**b**^ **(2.776)**	…	1.211 (2.776)	1.092 (2.776)	1.08 (2.776)	1.45 (2.776)	1.38 (2.776)	1.37 (2.776)	1.26 (2.776)	1.31 (2.776)	0.16 (2.776)	0.26 (2.776)	1.06 (2.776)	…	0.19 (2.776)	0.99 (2.776)	0.48 (2.776)	0.11 (2.776)	0.87 (2.776)	0.92 (2.776)	2.94 (2.776)

^a^ Principal com ponent regression (PCR) and partial least squares (PLS) chemometric methods

^b^The values are the corresponding tabulated values of t and F at p = 0.05

**Table 5:** Results of ANOVA (single factor) for comparison of the proposed methods and the reported methods for determination of TCB and LVM in pure powder form

	Source of Variation	Sum of squares	Degree of freedom	Mean square	F	F crit
**TCB**^**a**^ [5]	Between groups	3.248	7	0.464	0.422	2.249
	Within groups	43.963	40	1.099		
	Total	47.211	47			
**LVM**^**b**^ [22]	Between groups	3.379	3	1.12	0.797	3.098
	Within groups	28.24	20	1.41		
	Total	31.62	23			
Comparison with the reported method for determining TCB and LVM in Eradex Forte^®^ Suspension						
**TCB**^**c**^ [8]	Between groups	12.928	11	1.18	0.433	2.22
	Within groups	65.085	24	2.71		
	Total	78.013	35			
**LVM**^**c**^ [8]	Between groups	17.356	7	2.48	0.93	2.66
	Within groups	42.708	16	2.67		
	Total	60.06	23			

^a^ Spectrophotometric method using 0.1 M methanolic HCl as a solvent at wavelength of 305 nm

^b^ Spectrophotometric method using 0.1N HCl as a solvent at wavelength of 216 nm

^c^ Principal component regression (PCR) and partial least squares (PLS) chemometric methods

## Greenness assessment for the developed methods

### National environmental methods index (NEMI)

**Fig. 6:**
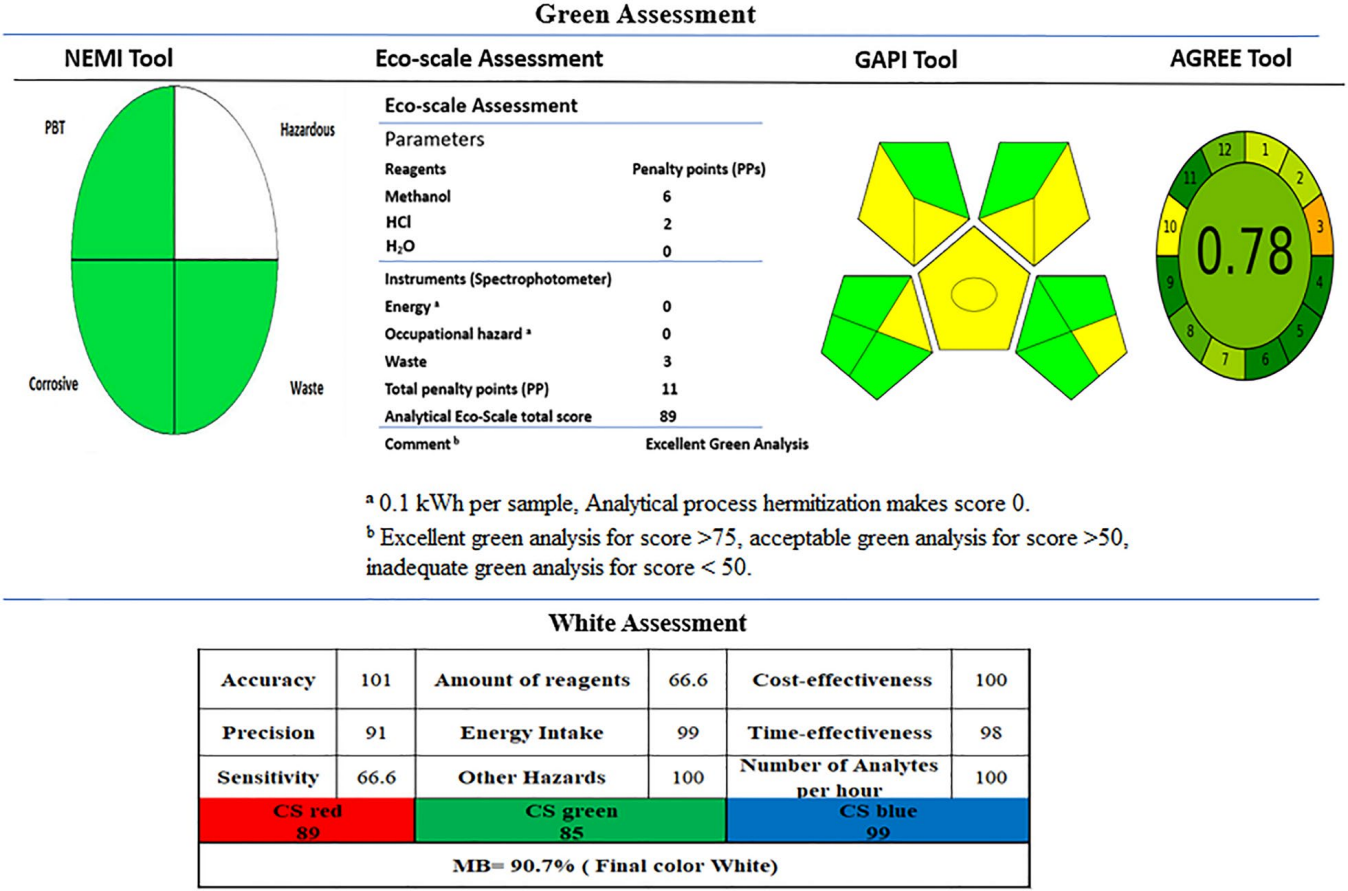
Greenness and White assessment of the proposed spectrophotometric methods

NEMI is regarded as one of the first methods for assessing green analytical chemistry. A circular diagram with four quadrants was constructed. Each one showed specific criteria including the usage of PBT (persistent, bioaccumulative and toxic) chemicals, consumption of hazardous chemicals, corrosiveness and the amount of waste generated [50, 51]. Three quadrants of the proposed methods showed green color as methanol doesn’t exist either in the PBT list or in the TRI (Toxic Release Inventory) list, the pH of the study samples was non-corrosive (pH is not less than 2 and not higher than 12) and the amount of waste produced was less than 50 g/sample. While the only blank quadrant due to the presence of methanol on the respective Resource Conservation and Recovery Acts lists as shown in Fig. 6.

### Analytical eco-scale system (AES)

It was proposed by [52], depending on subtraction of the penalty points (PPs) which represent the harmful impacts of the method on the environment from 100. The AES assessment results showed that methanol have 6 PPs as it has 3 pictograms with the signal word “Danger” and uses less than 10 mL of sample volume for analysis (sample cuvette). Also, HCl which exists in very low concentration has only one pictogram with 2 PPs and its volume consumption during sample analysis (sample cuvette) is less than 10 mL. While for the waste in the instrumentation has 3 PPs because of 1–10 mL of waste with recycling by distillation. According to this assessment tool, when the score > 75, it will be regarded as excellent green analysis. And when the final score is greater than 50, it will be regarded as acceptable green analysis. Inadequate green analysis is given for a final score less than 50. The AES assessment results are shown in Fig. 6 with excellent green analysis**.**

### Green analytical procedure index (GAPI)

The GAPI assessment tool provides both semi-quantitative information and primary basic information. It has the ability to assess the whole analytical procedure [53]. The GAPI pictogram has 5 main sections with 15 detailed parameters. The color of the pictogram parts may be green, yellow, and red where the green color indicates a safe procedure while the red refers to non-ecofriendly operations. The developed spectrophotometric methods have 8 green shaded sections and 7 yellow shaded sections with no red shading. Figure 6 shows the pentagrams of the proposed method having low environmental influence.

### Analytical greenness metric (AGREE)

Agree tool is a novel software which can be downloaded for green assessment [54]. It is considered a circle-colored pictogram with 12 principles of green analytical chemistry. Each section is colored from deep green (1) to deep red (0) depend on the environmental impact. The final score from 0 to 1 is displayed in the middle of that pictogram. All sections are colored deep green except Sect. 8 and 12 which are near deep green as the number of analytes in a single run are 2 with 20 samples per hour, methanol is considered highly inflammable respectively. While Sect. “Introduction”, “Experimental” and “Conclusion” have a light green color due to the at-line analysis of the sampling procedure, 1 mL amount of sample and the low volume of waste respectively. Section 10 has yellow color because some of reagents used are bio-based. Sect. “Results and discussion” has orange color as the at-line analytical device position. The proposed spectrophotometric methods AGREE score is (0.78) as shown in Fig. 6.

## White analytical chemistry tools (WAC)

The purpose of WAC is to examine the analytical techniques from a variety of angles, such as their effectiveness, their effects on environment, and their sustainability from an economical point of view [55]. The recently established Red–Green–Blue (RGB) model, a quantitative evaluation tool that enables sustainability by measuring the whiteness of the analytical method, makes available algorithms for global evaluations of analytical methods. The analytical data refer to red color includes accuracy, precision, linearity range and LOD. While the green color involves amount of reagents, amount of waste, toxicity of chemicals, energy consumption and additional occupational risks. The Blue color contains cost and time effectiveness, sample material consumption, instrument service frequency and risk of random instrumental malfunctions. A free excel sheet is used in the evaluation of WAC using RGB algorithm with a result out of 100. The total score of the whiteness for the proposed spectrophotometric methods is 90.7 as shown in Fig. 6.

## Conclusion

The three methods RS/SS, D^0^ CM/SS, and D^0^ FC/SS succeeded in obtaining each component spectrum alone in its D^0^ absorption spectrum with good accuracy and precision. The less extended component LVM was obtained by RS, and the extended component TCB was obtained by either D^0^ CM or D^0^ FC resolution techniques. Each time the resolved spectrum obtained was subtracted from the spectrum of the lab mixture to produce the D^0^ absorption spectrum of the other component by SS, that proved to be an excellent complementary method for each technique.

Similarly, the three methods DS/SS, D^1^ CM/SS, and D^1^ FC/SS succeeded to generate the spectrum of each component alone in its first derivative form with good accuracy and precision. The less extended component LVM was obtained by DS, and the extended component TCB was obtained by either D^1^ CM or D^1^ FC resolution techniques. Each time the resolved spectrum acquired was subtracted from the first derivative lab mixture spectrum to derive the first derivative spectrum of the other component by SS, that proved to be an excellent complementary method for each technique that allowed discarding the whole spectrum of the interfering substance.

The proposed methods showed their sensitivity and selectivity with few mathematical manipulation steps. Each spectrum was filtered alone to its D^0^ spectrum which performed as spectral profile of each cited drug allowed establishing spectra typical to the pure components present in Eradex Forte suspension^®^. In comparison to the CV method, there were very few manipulation steps in the concentration value method, as the concentration was shown graphically without substitution in regression equations. The proposed methods were determined with acceptable accuracy and precision at their maxima. The evaluation of the established procedures for the studied mixture's greenness and whiteness confirmed its safety, sustainability and improved time and cost effectiveness. The greenness of analytical procedures was assessed using the most efficient assessment tools GAPI and AGREE, which provided integrated data on the entire utilized technique. Compared to GAPI, AGREE make analysis completed in a short amount of time by the freely downloadable software. GAPI offers visual examination and an assessment of all the phases of an analytical process. ESA uses numbers to refer for the greenness so they can be utilized together as method evaluation tools. NEMI is the simplest and least efficient greenness tool and should be used in conjunction with other reliable assessment tools. As a result of varying detail levels for each element assessed, the tools are different from one another. As a result, the analyst must decide which metric will be most useful. The WAC is a simple tool to evaluate various analytical techniques including the effectiveness of the techniques, environmental impact, and economic considerations through a provided Excel work sheet. They could be strongly used in QC laboratories to regularly analyze the studied drugs in their pure bulk powders or dosage forms without the need for any prior separation steps.

## Data Availability

The datasets used and/or analysed during the current study are available from the corresponding author on reasonable request.

## Abbreviations

TCB: Triclabendazole
LVM: Levamisole
SS: Spectrum subtraction
RS-SS: Ratio subtraction method coupled spectrum subtraction
DS-SS: And derivative subtraction method coupled spectrum subtraction
CM: Constant multiplication
FC: Factorized response
DD^1^: Derivative ratio
CV: Constant value
CNV: Concentration value
NEMI: National Environmental Methods Index
AES: Analytical Eco-scale System
GAPI: Green Analytical Procedure Index
AGREE: Analytical greenness metric
WAC: White Analytical Chemistry tools
